# Characterization of arsenic species by liquid sampling-atmospheric pressure glow discharge ionization mass spectrometry

**DOI:** 10.1007/s00216-024-05312-x

**Published:** 2024-05-04

**Authors:** Joseph Goodwin, R. Kenneth Marcus, Garnet McRae, Ralph E. Sturgeon, Zoltan Mester

**Affiliations:** 1 Department of Chemistry, Clemson University, Clemson, SC 29634 USA; 2 Metrology Research Center, National Research Council Canada, Ottawa, Ontario K1A0R6 Canada

**Keywords:** Speciation, Glow discharge, Arsenic, Mass spectrometry, APGD

## Abstract

A liquid sampling-atmospheric pressure glow discharge (LS-APGD) ionization source operating at a nominal power of 30 W and solution flow rate of 30 µL min^−1^ and supported in a He sheath gas flow rate of 500 mL min^−1^ was interfaced to an Orbitrap mass spectrometer and assessed for use in rapid identification of inorganic and organic arsenic species, including As(III), As(V), monomethylarsonic acid, dimethylarsinic acid, and arsenobetaine in a 2% (v/v) nitric acid medium. Mass spectral acquisition in low-resolution mode, using only the ion trap analyzer, provided detection of protonated molecular ions for AsBet (*m/z* 179), DMA (*m/z* 139), MMA (*m*/*z* 141), and As(V) (*m/z* 143). As(III) is oxidized to As(V), likely due to in-source processes. Typical fragmentation of these compounds resulted in the loss of either water or methyl groups, as appropriate, i.e., introducing DMA also generated ions corresponding to MMA and As(V) as dissociation products. Structure assignments were also confirmed by high-resolution Orbitrap measurements. Spectral fingerprint assignments were based on the introduction of solutions containing 5 µg mL^−1^ of each arsenic compound.

## Introduction

Characterization of anthropogenic and biogenic metal and metalloid species in biological and environmental compartments is of substantial interest as their potential mobility, bioavailability, environmental behavior and toxicity are often highly dependent on their specific chemical forms and oxidation states [1]. The use of high-resolution chromatography coupled with element-specific detection systems (notably ICP-MS) has substantially advanced the state of speciation analysis; however, since identification often may rest solely on species retention times, issues arise when analyte calibration standards are not available to identify responses from unknown species present in complex (natural) samples [2–4]. Consequently, since the early days of speciation analysis, there have been ongoing efforts to apply unambiguous analytical strategies based on “organic” molecular mass spectrometry for the structural identification of analyte species. For volatile compounds, GC separations with traditional electron ionization (EI) mass spectrometry (MS) have been utilized [5, 6] or interfaced with external element-specific detection systems [7, 8], whereas electrospray ionization (ESI) MS has been the method of choice for analysis of targeted and non-targeted non-volatile, polar, or ionic molecular species [9, 10]. Despite a lack of comprehensive species-specific calibrants, identification issues can largely be obviated based on ESI coupled with high-resolution accurate mass spectrometry. Although not without issues (for quantitation), including matrix effects [11] and species-specific response [9, 12], this approach permits intact molecules to be targeted and identified based on their accurate mass or fragmentation signatures instead of detecting specific elements [13].

In addition to ESI strategies, there has been a growing interest in the development of complementary/alternative atmospheric pressure low temperature (soft) desorption/ionization sources [14]. Amongst these are commercially available direct analysis in real time (DART) and desorption electrospray ionization (DESI) devices, which are based on the exposure of analyte to the afterglow/ion plume of a (typically He) corona discharge or an electrospray source of highly charged aqueous aerosol to desorb and ionize analyte molecules from support surfaces, respectively [15, 16]. Using a DART ionization source, detection of B, As, Fe, Hg, Pb, Pd, Se, and Sn species has been reported [17–21]. DART has been coupled to a variety of separation techniques, including GC, HPLC, CE, and TLC, and these applications take advantage of this ion source being able to interrogate complex, multiphase systems originating from a chemical vapor generation process.

Several additional atmospheric pressure plasma sources capable of generating molecular ions and fragments useful for speciation analysis have been reviewed by Shelley et al. [22]. Of significant potential are atmospheric pressure glow discharge devices (APGD), such as the flowing atmospheric pressure afterglow (FAPA), suitable for the characterization of both gas phase and solid compounds [23, 24] as well as various embodiments of liquid-based atmospheric pressure glow discharges wherein the glow discharge plasma is established in the gap between the sample solution and an electrode under high voltage, with liquids effectively serving as one or both of the discharge electrodes. Both solution anode and solution cathode variants have been studied [25]. Based on the pioneering work of Cserfalvi and Mezei, who developed the electrolyte-cathode discharge [26], Webb et al. [27] subsequently simplified the device and termed it a solution cathode glow discharge (SCGD).

As early as 2001, Marcus and Davis [28] further refined this basic source to develop a true liquid LS-APGD microplasma operating in a total consumption mode (30–100 µL min^−1^), eventually realizing an efficient source for combined atomic and molecular (CAM) ionization yielding excellent performance in both modes [29–31]. When subsequently coupled to high-resolution Orbitrap instrumentation, the full potential of this microplasma device was realized [32, 33]. The latter publication highlighted the usefulness of the LS-APGD source for the production of molecular species of interest to the field of speciation. The degree of fragmentation (atomic *vs.* molecular) can be tuned via changes in the electrolyte composition, the discharge current, and the use of multiple counter electrodes [34].

Arsenic is amongst a growing set of elements (viz. mercury, selenium, tin, chromium, copper, sulfur, and phosphorus) that have received significant attention from the speciation community. The rich biochemistry of arsenic, especially in marine environments, is well appreciated as it is present not only as water-soluble but also lipophilic compounds [35]. Apart from the studies by Hoegg et al. [33] and *Zhang *et al*.* [36], very few additional applications of the LS-APGD source for elemental speciation have been published to date. Herein, we provide the first preliminary report on the characterization of the LS-APGD source coupled to an ion trap mass spectrometer for the rapid identification of a simple suite of low molecular weight arsenic species, including arsenous acid [As(III)], arsenic acid [As(V)], monomethylarsonic acid (MMA), dimethylarsinic acid (DMA), and arsenobetaine (AsBet) presented in an electrolyte medium of 2% (*v*/*v*) nitric acid. Since a dilute nitric acid electrolyte is used as the conductive medium, it is evident that the protonated species of these compounds will be present in the test solutions. Given the LS-APGD’s unique ability to act as a CAM ionization source, information was obtained from both inorganic and organic arsenic species in a single analysis, something which is hampered by low sensitivity with traditional ionization sources coupled to liquid-based mass spectrometric platforms (i.e., ESI). In addition to providing more coverage of the possible inorganic and organoarsenic compounds, the low power and gas requirements of the LS-APGD make it an ideal source for field-portable mass spectrometers, resulting in an analytical platform capable of delivering rapid, high-quality, on-site analysis for data-driven studies in the field.

## Experimental

### Instrumentation

#### Liquid sampling-atmospheric pressure glow discharge (LS-APGD)

**Fig. 1:**
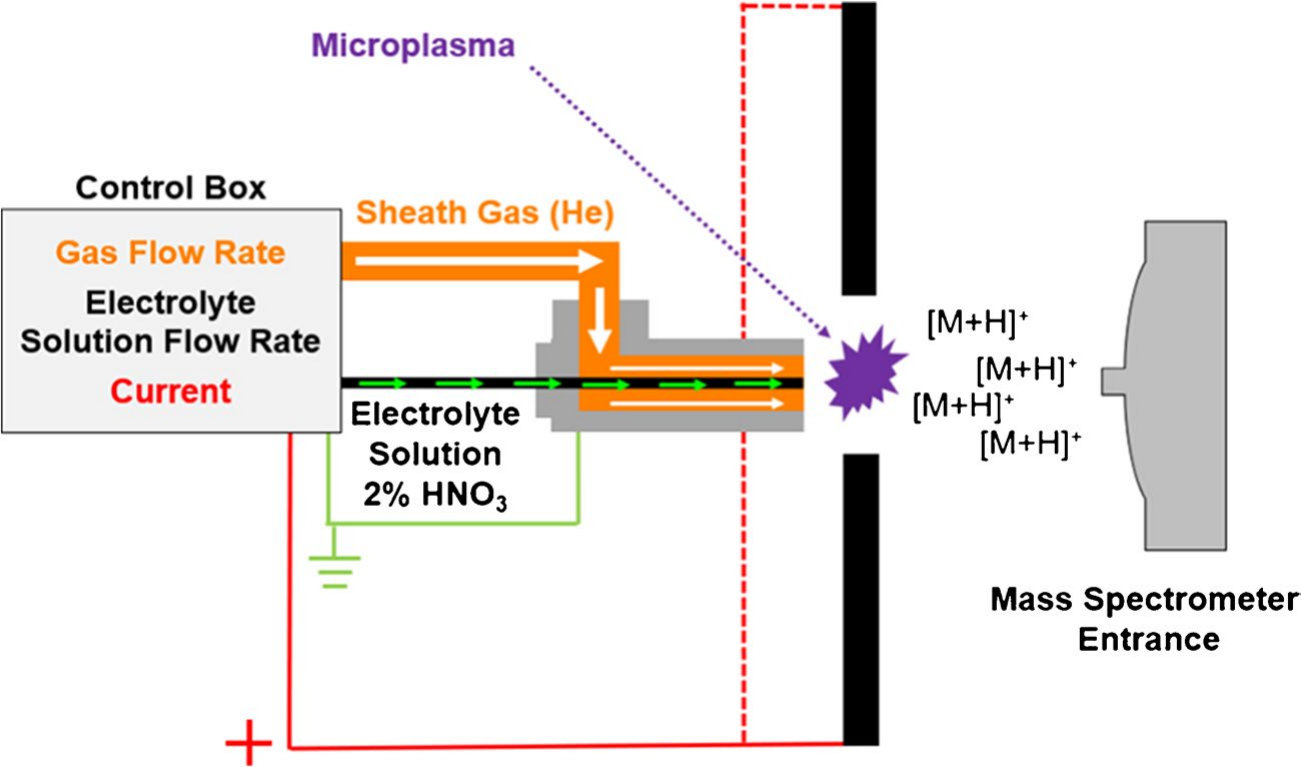
Schematic of major components of the LS-APGD ion source

A dual-electrode LS-APGD ionization source, described previously [37] and graphically depicted in Fig. 1, was used for all measurements. This ionization source consists of two stainless steel powered anode electrodes (weldable feedthrough, MDC Vacuum Products, LLC, Hayward, CA) and a grounded solution cathode. The solution cathode comprises two components: an outer stainless steel tube (0.04 in I.D. × 1/16 in O.D.; McMaster-Carr, Elmhurst, IL) that directs a helium sheath gas to the plasma and a concentric inner silica capillary (250 μm I.D. × 360 μm O.D.; Molex, Lisle, IL) that delivers the electrolyte (sample) solution to the plasma. The discharge is sustained between both powered anodes and the grounded solution cathode. A control module, designed and built by GAA Custom Electronics (Kennewick, WA), was used to supply power and adjustable solution flow to the plasma discharge. A six-port, two-position Rhedoyne valve was equipped with a 20-µL sample loop. A solution flow rate of 30 µL min^−1^, a He sheath gas flow rate of 500 mL min^−1^, an inter-electrode gap of 1.5 mm, and a discharge current of 30 mA at a discharge voltage of approximately 500 V were used for all measurements.

**Fig. 2:**
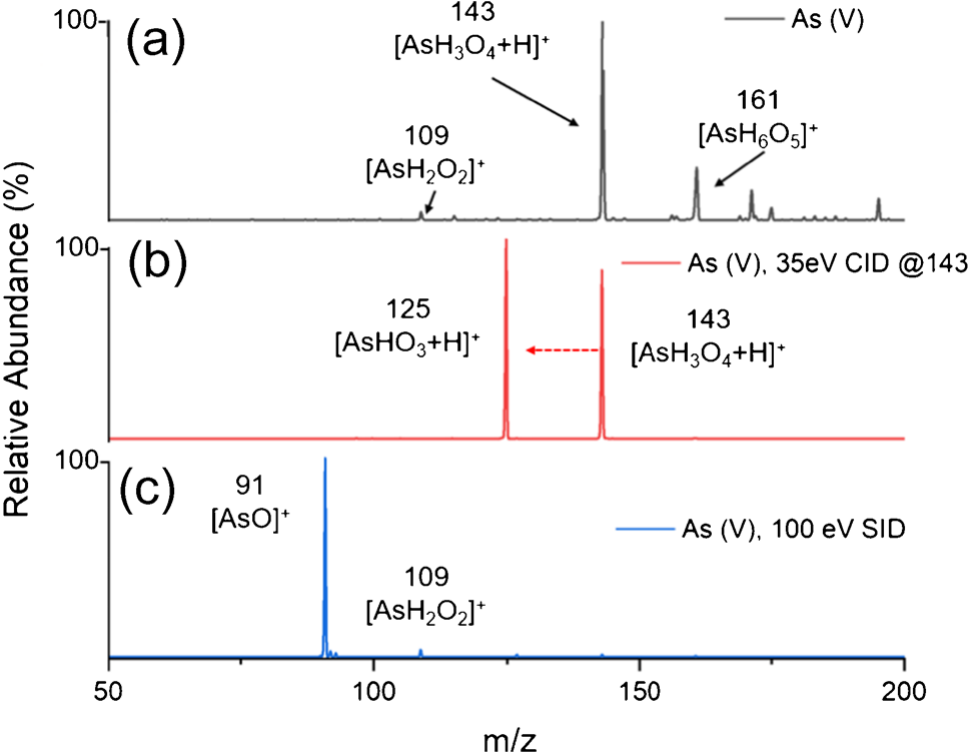
Mass spectral scan of 100 ng As(V). **a** Full scan of As(V) without CID or SID. **b** Fragmentation of protonated As(V) after isolation and application of 35 eV CID. **c** Fragmentation pattern after application of 100 eV of SID to the full injection transient

A Thermo LTQ Orbitrap was used with no modifications other than the removal of the manufacturer’s ionization source (ESI) and its replacement with the LS-APGD ionization source. Only the quadrupole ion trap analyzer was used to generate mass spectral transients with a typical ion injection time of 10 ms and 10 microscans per scan. Mass ranges of 50–200 *m/z* or 50–150 *m/z* were used for most measurements. After identification of the protonated molecular ion for organic arsenic species, the protonated ion was isolated (width of 1 Da) and fragmented using 35 eV of collisional induced dissociation (CID) in the ion trap. Additionally, 100 eV of in-source collisional induced dissociation (SID) was used in the high-pressure region of the sampling interface to generate fragmentation spectra shown in Fig. 2c and Fig. 7.

### Reagents

Arsenic(V) oxide (99%), arsenic(III) oxide (99.995%), monomethylarsonic acid (99%) (MMA), and dimethylarsinic acid (99.0%) (DMA) were purchased from Sigma-Aldrich (St. Louis, MO). Reference material ABET-1 (National Research Council Canada, Ottawa) served as the source for arsenobetaine (AsBet). Table 1 summarizes these analyte species and their chemical structure. Test solutions (5 µg mL^−1^ (as As) for each compound) were prepared in 2% (*v*/*v*) nitric acid, which has historically been used as an electrolyte solution for elemental analysis with the LS-APGD, given the solubility of nitrate salts [32]. In conjunction with a 20-µL injection loop, analytical response arises from 100 ng total analyte (as arsenic) delivered to the LS-APGD.

**Table 1:**
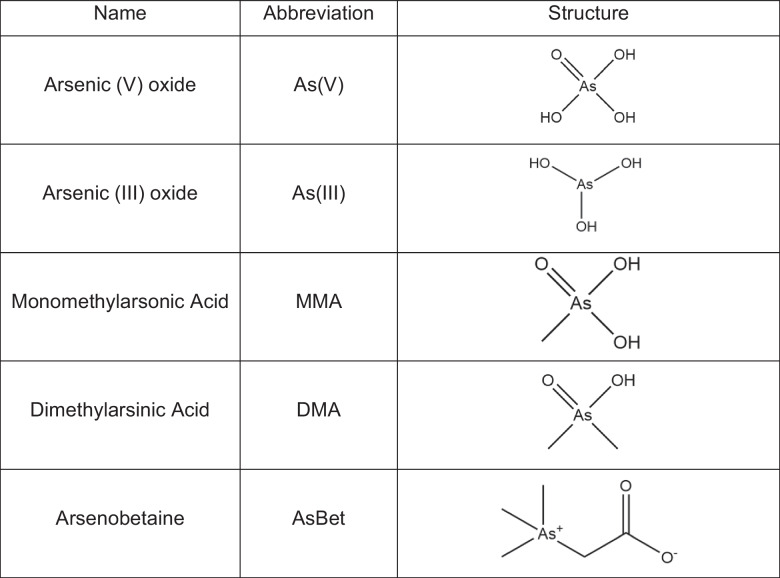
Arsenic species examined and their structure

## Results

### Inorganic arsenic species

#### Arsenic(V)

Delivery of As(V) to the LS-APGD microplasma ionization source operated in positive MS ion mode results in the detection of the protonated molecular ion at *m/z* 143 [AsH_3_O_4_ + H]^+^. As shown in Fig. 2a, a peak at *m/z* 161 was also observed, likely resulting from the addition of water to the protonated molecular ion. When the protonated molecular ion at *m/z* 143 was subjected to 35 eV CID, the loss of water yielded a peak at *m/z* 125 [AsHO_3_ + H]^+^, as shown in Fig. 2b. By applying 100 eV SID, a peak at *m/z* 91, corresponding to [As = O]^+^, as well as a peak at *m/z* 109 arising from the latter’s association of a water molecule ([As H_2_O_2_]^+^) were detected, as evident in Fig. 2c.

#### Arsenic(III)

**Fig. 3:**
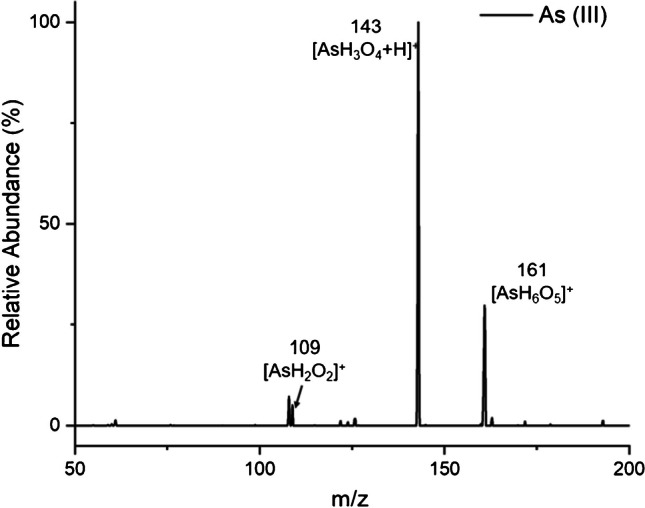
Mass spectral scan of 100 ng As(III). The spectrum is similar to that obtained for As(V) in Fig. 2a

No significant difference is evident between the mass spectrum arising from the introduction of a 20 µL solution of 5 µg mL^−1^ As(III) and that from 5 µg mL^−1^ As(V), as shown in Fig. 3, with the primary peaks observed at *m/z* 143 being the same as the protonated molecular ion for As(V). It is suspected that this is due to in-source oxidative processes. Brief studies (not shown) were conducted using a solution of methanol and water (70:30 *v/v* respectively), which also showed similar spectra between As(V) and As(III), which suggests the similarity in spectra is not related to the electrolyte solution, but perhaps due to gas-phase reactions with radical oxygen species. The same protonated molecular ion at *m/z* 143 and fragments and adducts at 161, 125, and 109 are also evident. While having the same spectral signatures can be thought of as problematic, these two inorganic arsenic species are readily separated chromatographically [38] and themselves have distinct spectral features versus organoarsenic species.

### Organoarsenic species

#### Monomethylarsonic acid (MMA)

**Fig. 4:**
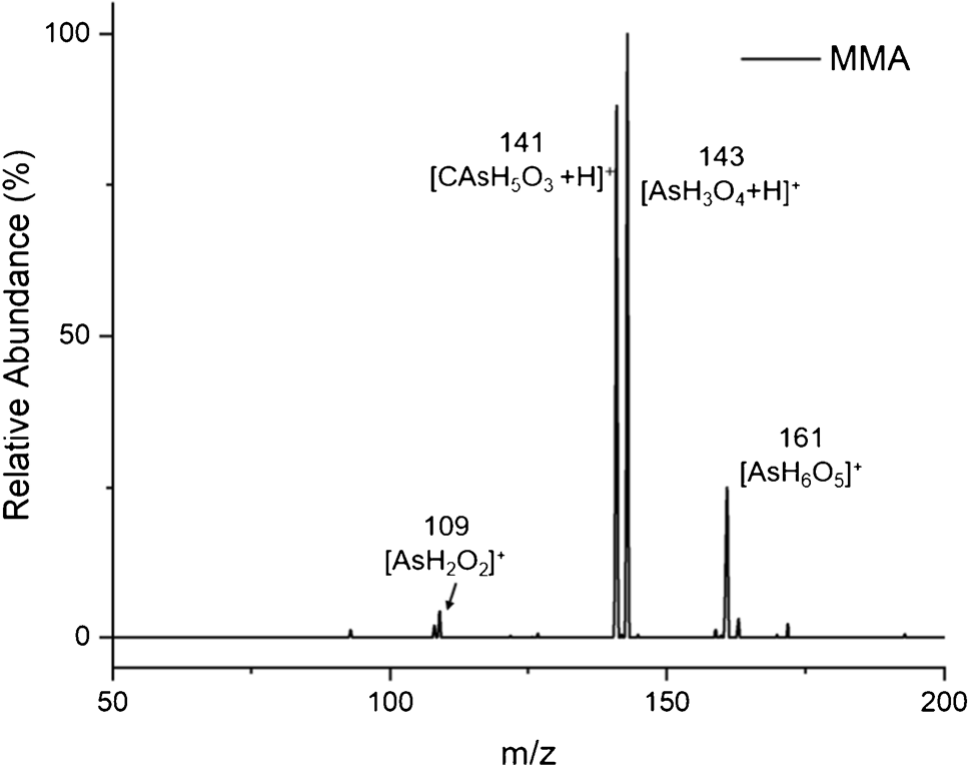
Mass spectral scan of 100 ng As as MMA. A unique signature was generated at *m/z* 141 representing protonated MMA

A protonated molecular ion of MMA at *m/z* 141 [CAsH_5_O_3_ + H]^+^ was detected using LS-APGD, as shown in Fig. 4. Additionally, peaks at *m/z* 143, 161, and 109 are also present, common to those found from the inorganic As(V)/As(III) species noted earlier. When the protonated molecular ion was subjected to 35 eV CID, a peak at *m/z* 123 was observed, indicating a similar water loss pattern as noted for As(V) in Fig. 2b. Interestingly, introducing a standard MMA solution generates significant signals corresponding to those characteristics of inorganic arsenic. Although solutions of MMA were verified as being free of inorganic arsenic, signal intensities at *m*/*z* 143 (inorganic arsenic) and *m*/*z* 141 (protonated MMA) are similar. The presence of inorganic arsenic peaks is likely due to systematic demethylation/oxidation of MMA in the relatively energetic source.

#### Dimethylarsinic acid (DMA)

**Fig. 5:**
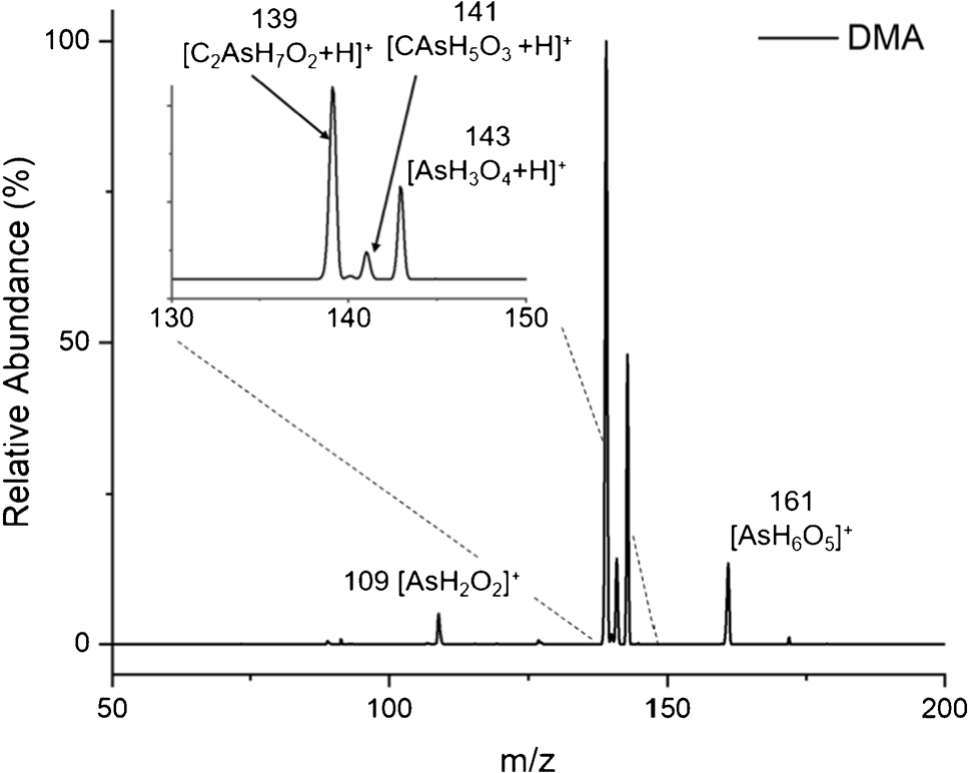
The mass spectral scan of 100 ng As as DMA generated a unique signature at *m*/*z* 139 representing protonated DMA. The inset shows the expanded region between 130 and 150 Da

The mass spectrum arising from DMA is shown in Fig. 5, revealing a unique peak at *m/z* 139 arising from the protonated molecular ion [C_2_AsO_2_H_7_ + H]^+^. Additionally, peaks at *m/z* 141 [CAsH_5_O_3_ + H]^+^ characteristic of MMA, as well as *m/z* 143, 161, and 109, are common to those found for inorganic arsenic. As with MMA, the presence of characteristic inorganic arsenic signatures is suspected to be due to demethylation/oxidation in the ion source. As with MMA and inorganic arsenic, subjecting the protonated molecular ion (*m/z* 139) of DMA to 35 eV CID resulted in fragments corresponding to the loss of water (not shown).

#### Arsenobetaine (AsBet)

**Fig. 6:**
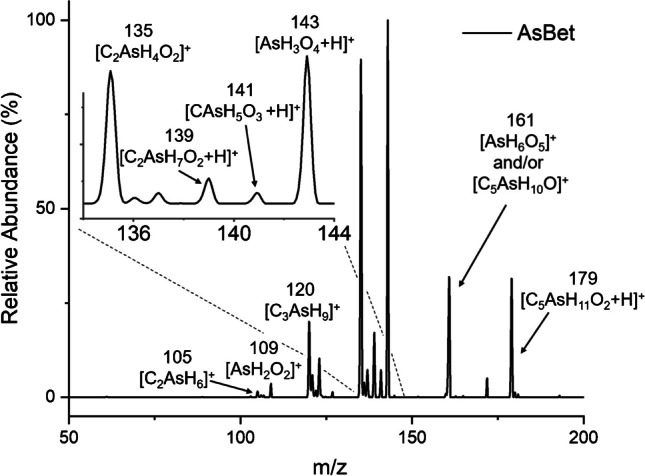
Mass spectral scan of 100 ng As as AsBet showing unique fragments at *m*/*z* 179 (representing protonated AsBet) as well as at 135, 120, and 105. The inset shows an expanded view between 134 and 144 Da

In common with other organic arsenic species, the introduction of AsBet produced a unique protonated molecular ion at *m/z* 179 [C_5_AsH_11_O_4_ + H]^+^, as shown in Fig. 6. In addition, other *unique* fragments are also present at *m/z* 135, corresponding to [C_2_AsH_4_O_2_]^+^ and 120, as [C_3_AsH_9_]^+^. The peak observed at *m/z* 120 was previously reported as an AsBet CID fragment with ESI-MS [39]. A small peak appears at *m/z* 105, which is consistent with [C_2_AsH_6_]^+^, a previously reported AsBet CID ESI–MS fragment used to confirm the presence of a trimethylarsonio moiety in an unknown organic arsenic species detected in coral reef fish [40]. In addition, a CID ESI–MS fragment at 105 has also been reported for arsenocholine, adding further to the use of this peak as a marker for trimethylarsonio organoarsenic compounds [39]. The presence of the *m/z* 105 peak illustrates the LS-APGD’s ability to provide fragmentation indicative of the molecular formula without using a subsequent fragmentation operation further along the ion beam path, such as CID. A peak appears at *m/z* 139 [C_2_AsO_2_H_7_ + H]^+^ as seen in DMA, and a peak at 141 [CAsH_5_O_3_ + H]^+^ as seen for MMA. In addition, the “inorganic peaks” at *m/z* 143, 161, and 109 are also present. Identification of the peak at *m/z* 161 is complicated by studies performed applying 35 eV of CID to an isolated moiety at *m/z* 179 (not shown). This process resulted in loss of water from protonated AsBet at *m/z* 161 [C_5_AsH_10_O]^+^, a fragment previously reported by McSheehy et al. during fragmentation studies using ESI-MS [41]. Given that other peaks indicative of inorganic arsenic are present without fragmentation, it is likely that the peak at *m/z* 161 is common to those found for inorganic arsenic. The peak at 161 possibly reflects the loss of water from AsBet as well as AsH_6_O_5_^+^.

### Peak assignment summary

Table 2 summarizes characteristic peaks present in the mass spectra along with their likely assignments. It is evident that As(III) and As(V) are indistinguishable, likely due to the oxidizing environment provided by the ion source. However, *unique* protonated molecular ions are generated for MMA, DMA, and AsBet at *m*/*z* 141, 139, and 179, respectively, as well as at *m/z* 105, 120, and 135, arising from the fragmentation of AsBet. Most structure assignments were corroborated by high-resolution orbitrap-MS measurements. Based on the presence of these unique peaks, it is clear that the LS-APGD source can ionize organoarsenic species, resulting in mass spectra that can be used for qualitative identification of unknown arsenic compounds.

**Table 2:** Characterization of inorganic and organic arsenic species

Compound	*m*/*z*	Elemental formula
As(V)	109	[AsH_2_O_2_]^+^
	143	[AsH_3_O_4_ + H]^+^ Protonated As(V) molecular ion
	161	[AsH_6_O_5_]^+^
As(III)	109	[AsH_2_O_2_]^+^
	143	[AsH_3_O_4_ + H]^+^
	161	[AsH_6_O_5_]^+^
MMA	109	[AsH_2_O_2_]^+^
	141	[CAsH_5_O_3_ + H]^+^ Protonated MMA molecular ion
	143	[AsH_3_O_4_ + H]^+^
	161	[AsH_6_O_5_]^+^
DMA	109	[AsH_2_O_2_]^+^
	139	[C_2_AsH_7_O_2_ + H]^+^ Protonated DMA molecular ion
	141	[CAsH_5_O_3_ + H]^+^
	143	[AsH_3_O_4_ + H]^+^
	161	[AsH_6_O_5_]^+^
AsBet	105	[C_2_AsH_6_]^+^
	109	[AsH_2_O_2_]^+^
	120	[C_3_AsH_9_]^+^
	135	[C_2_AsH_4_O_2_]^+^
	139	[C_2_AsH_7_O_2_ + H]^+^
	141	[CAsH_5_O_3_ + H]^+^
	143	[AsH_3_O_4_ + H]^+^
	161	[AsH_6_O_5_]^+^
		[C_5_AsH_10_O]^+^
	179	[C_5_AsH_11_O_2_ + H]^+^ Protonated AsBet molecular ion

### In-source collisional induced dissociation (SID)

**Fig. 7:**
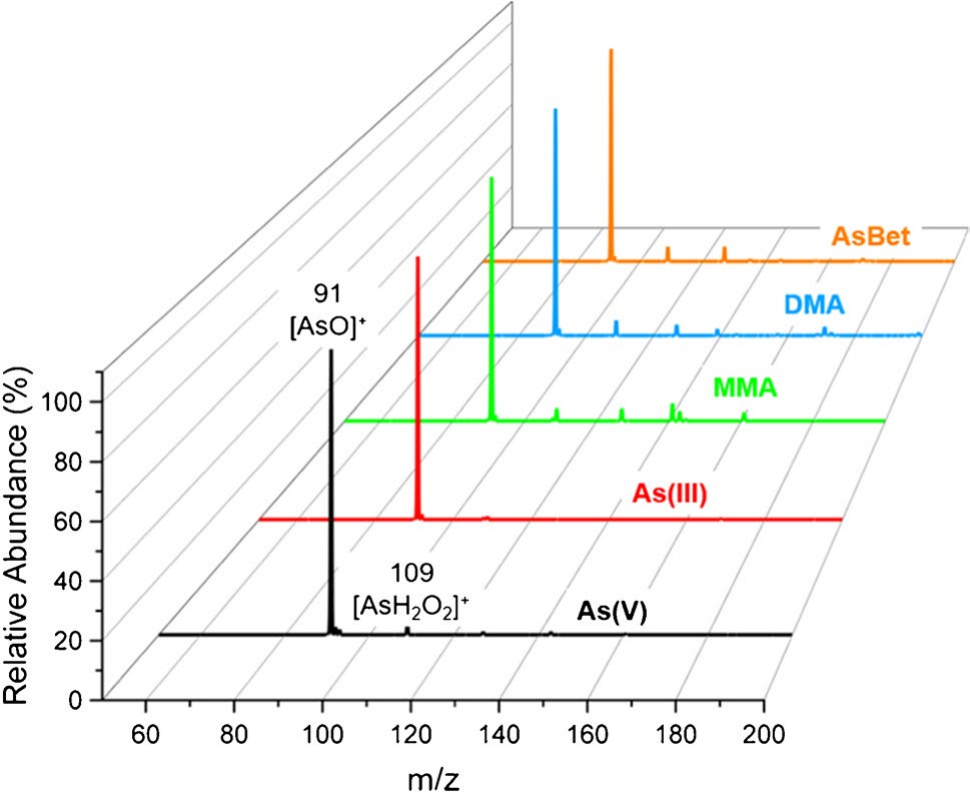
Effect of 100 eV SID on targeted arsenic species. The main peak arising for all compounds at *m/z* of 91 illustrates that high levels of SID can be used to reduce organic species and provide an estimate of total arsenic

In an attempt to illustrate the potential of the LS-APGD to serve as a species independent ion source for detection of total arsenic content in a manner similar to ICP-MS, the impact of 100 eV of SID was examined across the test species. All inorganic and organic arsenic compounds tested fragmented to yield a base peak at *m/z* 91, as shown in Fig. 7. This peak, evident in both spectra generated from inorganic As and organic As under the same conditions, is assigned as [As = O]^+^. McSheehy et al. also noted this peak in a time-of-flight mass spectrum generated during ESI–MS interrogation of DMA but, within the uncertainty of the mass assignment, could not unequivocally discern between [CAsH_4_]^+^ and [As = O]^+^ [41]. To rule out an assignment of [CAsH_4_]^+^ for the organoarsenic compounds tested, a spectrum of both AsBet and As(V) with 100 eV of SID was collected with the ion trap in high-resolution mode (ultrazoom), which resulted in a peak within 0.01 Da for the peak at *m/z* 91 for both compounds, indicating that the peak is likely the same between the two compounds. Given the highly oxidative environment of the microplasma evident throughout these studies, an assignment of [As = O]^+^ is more probable; however, additional studies are necessary to draw a definitive conclusion. Based on the similar fragmentation pattern seen for both inorganic and organoarsenic compounds, it may be possible to perform a total arsenic screening analysis without prior knowledge of the possible constituents of the sample. Since dissociation is occurring in the source, no prior knowledge is necessary, i.e., a unique peak does not need to be isolated and fragmented to provide a characteristic, arsenic-indicating signature for all the organometallic arsenic species tested.

### Estimated limit of detection

As the primary focus of this study was a qualitative characterization of the LS-APGD source for arsenic speciation and not quantitative determinations, calibration curves and low-concentration injections were not examined. However, estimates of the limits of detection (LOD) in the low 10’s of ng mL^−1^ range (as arsenic) for the targeted species were obtained based on Eq. 1. These LODs compare favorably to those reported for organic arsenic with ESI sources [42, 43]. It is worth noting that these results were generated without fully optimizing plasma discharge conditions and mass spectrometer parameters which could result in substantial improvements in detection limits.

1 $${\text{LOD}}= \frac{3\times {\sigma }_{\mathrm{blank }}\times \mathrm{Sample Concentation as As }(\mathrm{ng }{{\text{mL}}}^{-1})}{\mathrm{Protonated Peak Height}}$$

## Discussion

Structural/species assignments described above were based on unit resolution ion trap MS analyses supplemented by relevant published literature on the MS detection of these compounds. The experimental design and objectives presented herein are quite simplistic. Clearly, HPLC can be readily utilized as the sample introduction methodology compatible with the operation of this LS-APGD. Thus, matrix-free (minimized) elution of (extracted) arsenic species can be achieved, which, at the least, mimics the performance of the limited suite of small molecule calibration solution standards individually characterized in this study. As this CAM source is shown to provide molecular information, it potentially straddles the realm of HPLC/ICP MS, wherein the ICP source destroys all speciation information and identification relies on peak retention times and matching standards, and that of ESI–MS, which potentially provides molecular species structural identification. It is thus interesting to compare the performance of the LS-APGD with that achieved using complimentary ESI sample introduction techniques. Unfortunately, reports on small molecule arsenic species detected by MS using atmospheric pressure ion sources are scarce [44]. A critical review by Reimer et al. [3] provides a succinct summary of the advantages and disadvantages of ESI, which are not repeated here, and published examples of ESI–MS spectra of these arsenic species.

ESI may be operated at both low (soft) and high (hard) energies to produce protonated molecular species and bare As^+^ ions from the arsenicals, respectively, permitting not only As-containing peaks to be identified but molecular mass information and, hence, structural identification of peaks to be assigned. The former is only possible when the effort is made to limit trace oxygen impurity in the CID gas to prevent the formation of otherwise dominant AsO^+^ [45]. Likely, for this reason, no bare As^+^ is evident in any of the LS-APGD spectra obtained with 100 eV SID in this study, only AsO^+^.

In an early study by Pedersen and Francesconi [46], it was noted that an LC-ESI MS approach successful for the detection of several organoarsenic compounds was not suitable for either inorganic arsenite or arsenate, but no discussion of this conclusion was presented. Shortly thereafter, however, the study by McSheehy et al. [47] is one of the few to discuss an ESI MS spectrum of As(V) present in numerous marine arsenosugar compounds in an extract of algae. Noteworthy is that the LS-APGD source produces the same protonated molecular ion peak at *m*/*z* 143 in addition to those at 109 and 161, also common to As(III) in this source. 100 eV SID of As(V) generates fragmentation products at *m*/*z* 91 and 109 and CID of *m/z* 143 at 35 eV generates fragmentation products at 125 similar to the fragmentation products produced with 35 eV CID using ESI MS [47]. Note that the CID energy to produce the most intense signal for a single fragment was not optimized, and thus, direct comparison of the relative intensities of LS-AGPD derived CID spectra may not reflect those evident in published ESI MS spectra.

Larsen et al. provide the ESI MS spectra of several of the protonated organoarsenical compounds common to this study [42]. High-energy ESI spectra can be acquired without significantly diminishing the base peaks of either DMA at *m*/*z* 139 or for MMA at *m*/*z* 141 [42]. ESI MS of AsBet generates unique peaks at *m*/*z* 179, 135, and 120, in common with those obtained with the LS-APGD source. Similarly, MMA and DMA are dominated by their protonated molecular ions at *m*/*z* 141 and 139, respectively, also mirrored by the results obtained with the LS-APGD source. No data were presented for As(III/V). Inoue et al. [43] present ESI MS spectra for MMA, DMA, and AsBet that are characterized by base peaks at *m*/*z* 141, 139, and 179, respectively, in agreement with those reported with LS-APGD in this study.

Detection of arsenic oxyanions is not particularly sensitive with ESI MS, making alternative ionization approaches of great interest. In this study, the LS-APGD ion source provided estimated detection limits (for arsenic) in the 10’s of ng mL^−1^ range, comparable to those achieved with ESI [42, 43]. However, given the focus of the present report, further studies are needed to achieve a more definitive assessment of quantitative measurement capabilities using the LS-APGD. At this point, it is important to reiterate that no attempts to optimize the plasma discharge conditions or the mass spectrometer parameters were made during this investigation. Further improvements in detection capability are expected when measurement parameters are fully optimized.

## Conclusions

Speciation capabilities for several small molecular arsenic species were examined using a novel LS-APGD ion source in combination with mass spectrometry detection. Estimates of detection limits for MMA, DMA, and AsBet were comparable to those of typical ESI MS approaches, while also effectively ionizing inorganic arsenic species, offering an attractive alternative for these organometallics. The APGD source appears to be significantly more energetic than ESI, and a degree of in-source oxidation and demethylation was often observed. For molecular/structural studies, a balance between response and oxidation must be considered.

Mass spectral fragmentation patterns of the species studied here were largely in accord with published ESI studies with the caveat concerning tunability properties of the LS-APGD source to elicit fragmentation during ionization. While not thoroughly evaluated in this study, operation of the LS-APGD at high power results in more pronounced fragmentation of the arsenic species. Previous studies with the LS-APGD have shown the source to be robust and able to operate with samples containing organic solvents and high salt contents, which typically present a challenge for ESI or even ICP sources. However, further studies are necessary to evaluate the LS-APGD’s ability to analyze arsenic-containing species thoroughly. Although this work only provides a first step towards sensitive discrimination of inorganic and organic arsenic species on one platform, it does show that concurrent analysis is feasible. In addition, this work builds upon previous studies that illustrate the opportunities that CAM ionization offers for challenging matrices [48] and metal speciation analyses. Overall, the performance of the LS-APGD suggests it may be a competitive ion source option for complex organometallic/speciation problems. Noteworthy is that estimated detection limits for these arsenic species are in the low 10s of ng mL^−1^ range (as arsenic) for both inorganic and organoarsenic species, which is difficult to achieve using conventional ESI sample introduction.

## References

[1] Templeton DM, Ariese F, Cornelis R, Danielsson L-G, Muntau H, van Leeuwen HP, Lobinski R. Guidelines for terms related to chemical speciation and fractionation of elements. Definitions, structural aspects, and methodological approaches (IUPAC Recommendations 2000). 2000;72(8):1453–70.

[2] Szpunar J, Lobinski R, Prange A (2003). Hyphenated techniques for elemental speciation in biological systems. Appl Spectrosc.

[3] Nearing MM, Koch I, Reimer KJ (2014). Complementary arsenic speciation methods: a review. Spectrochim Acta Part B At Spectrosc.

[4] LeBlanc KL, Mester Z. Compilation of selenium metabolite data in selenized yeasts. Metallomics. 2021;13(6). 10.1093/mtomcs/mfab031 34156080

[5] Ali I, Aboul-Enein HY. Instrumental methods in metal ion speciation: CRC Press; 2006.

[6] Yang L, Colombini V, Maxwell P, Mester Z, Sturgeon RE (2003). Application of isotope dilution to the determination of methylmercury in fish tissue by solid-phase microextraction gas chromatography–mass spectrometry. J Chromatogr A.

[7] Bouyssiere B, Szpunar J, Lobinski R (2002). Gas chromatography with inductively coupled plasma mass spectrometric detection in speciation analysis. Spectrochim Acta Part B At. Spectrosc.

[8] Queipo-Abad S, González PR, Martínez-Morillo E, Davis WC, García Alonso JI (2019). Concentration of mercury species in hair, blood and urine of individuals occupationally exposed to gaseous elemental mercury in Asturias (Spain) and its comparison with individuals from a control group formed by close relatives. Sci Total Environ.

[9] Feldmann J, Raab A, Krupp EM (2018). Importance of ICPMS for speciation analysis is changing: future trends for targeted and non-targeted element speciation analysis. Anal Bioanal Chem.

[10] Yang L, Ding J, Maxwell P, McCooeye M, Windust A, Ouerdane L (2011). Determination of arsenobetaine in fish tissue by species specific isotope dilution LC-LTQ-orbitrap-MS and standard addition LC-ICPMS. Anal Chem.

[11] Mallet CR, Lu Z, Mazzeo JR (2004). A study of ion suppression effects in electrospray ionization from mobile phase additives and solid-phase extracts. Rapid Commun Mass Spectrom.

[12] Indelicato S, Bongiorno D, Ceraulo L. Recent approaches for chemical speciation and analysis by electrospray ionization (ESI) mass spectrometry. Front Chem. 2021;8. 10.3389/fchem.2020.625945 PMC7855954 33553108

[13] Bierla K, Chiappetta G, Vinh J, Lobinski R, Szpunar J. Potential of fourier transform mass spectrometry (Orbitrap and Ion Cyclotron Resonance) for speciation of the selenium metabolome in selenium-rich yeast. Front Chem. 2020;8. 10.3389/fchem.2020.612387 PMC7755988 33363115

[14] Venter A, Nefliu M, Graham Cooks R (2008). Ambient desorption ionization mass spectrometry. TrAC, Trends Anal Chem.

[15] Chernetsova ES, Morlock GE (2011). Ambient desorption ionization mass spectrometry (DART, DESI) and its bioanalytical applications. Bioanal Rev.

[16] Gross JH (2014). Direct analysis in real time—a critical review on DART-MS. Anal Bioanal Chem.

[17] Borges DLG, Sturgeon RE, Welz B, Curtius AJ, Mester Z (2009). Ambient mass spectrometric detection of organometallic compounds using direct analysis in real time. Anal Chem.

[18] Vyhnanovský J, Kratzer J, Benada O, Matoušek T, Mester Z, Sturgeon RE (2018). Diethyldithiocarbamate enhanced chemical generation of volatile palladium species, their characterization by AAS, ICP-MS, TEM and DART-MS and proposed mechanism of action. Anal Chim Acta.

[19] Pagliano E, Onor M, McCooeye M, D’Ulivo A, Sturgeon RE, Mester Z (2014). Application of direct analysis in real time to a multiphase chemical system: identification of polymeric arsanes generated by reduction of monomethylarsenate with sodium tetrahydroborate. Int J Mass Spectrom.

[20] D’Ulivo L, Pagliano E, Onor M, Mester Z, D’Ulivo A (2019). Application of direct analysis in real time to the study of chemical vapor generation mechanisms: identification of intermediate hydrolysis products of amine-boranes. Anal Bioanal Chem.

[21] Matoušek T, Kratzer J, Sturgeon RE, Mester Z, Musil S (2021). A mass spectrometric study of hydride generated arsenic species identified by direct analysis in real time (DART) following cryotrapping. Anal Bioanal Chem.

[22] Shelley JT, Badal SP, Engelhard C, Hayen H (2018). Ambient desorption/ionization mass spectrometry: evolution from rapid qualitative screening to accurate quantification tool. Anal Bioanal Chem.

[23] Andrade FJ, Shelley JT, Wetzel WC, Webb MR, Gamez G, Ray SJ, Hieftje GM (2008). Atmospheric pressure chemical ionization source. 1. Ionization of compounds in the gas phase. Anal Chem.

[24] Andrade FJ, Shelley JT, Wetzel WC, Webb MR, Gamez G, Ray SJ, Hieftje GM (2008). Atmospheric pressure chemical ionization source. 2. Desorption−ionization for the direct analysis of solid compounds. Anal Chem.

[25] Liu X, Zhu Z. Plasma-mediated vapor generation techniques. In: D'Ulivo A, Sturgeon RE, editors. Vapor Generation Techniques for Trace Element Analysis: Elsevier; 2022. pp. 283–315.

[26] Cserfalvi T, Mezei P (1994). Direct solution analysis by glow discharge: electrolyte-cathode discharge spectrometry. J Anal At Spectrom.

[27] Webb MR, Andrade FJ, Gamez G, McCrindle R, Hieftje GM (2005). Spectroscopic and electrical studies of a solution-cathode glow discharge. J Anal At Spectrom.

[28] Marcus RK, Davis WC (2001). An atmospheric pressure glow discharge optical emission source for the direct sampling of liquid media. Analytical Chemistry.

[29] Marcus RK, Quarles CD, Barinaga CJ, Carado AJ, Koppenaal DW (2011). Liquid sampling-atmospheric pressure glow discharge ionization source for elemental mass spectrometry. Anal Chem.

[30] Zhang LX, Marcus RK (2016). Mass spectra of diverse organic species utilizing the liquid sampling-atmospheric pressure glow discharge (LS-APGD) microplasma ionization source. J Anal At Spectrom.

[31] Marcus RK, Manard BT, Quarles CD (2017). Liquid sampling – atmospheric pressure glow discharge (LS-APGD) microplasmas for diverse spectrochemical analysis applications. J Anal At Spectrom.

[32] Kenneth Marcus R, Hoegg ED, Hall KA, Williams TJ, Koppenaal DW (2023). Combined atomic and molecular (CAM) ionization with the liquid sampling-atmospheric pressure glow discharge microplasma. Mass Spectrom Rev.

[33] Hoegg ED, Godin S, Szpunar J, Lobinski R, Koppenaal DW, Marcus RK (2019). Ultra-high resolution elemental/isotopic mass spectrometry (m/Δm > 1,000,000): coupling of the liquid sampling-atmospheric pressure glow discharge with an orbitrap mass spectrometer for applications in biological chemistry and environmental analysis. J Am Soc Mass Spectr.

[34] Williams TJ, Marcus RK (2020). Coupling the liquid sampling – atmospheric pressure glow discharge, a combined atomic and molecular (CAM) ionization source, to a reduced-format mass spectrometer for the analysis of diverse species. J Anal At Spectrom.

[35] Frankenberger Jr WT. Environmental chemistry of arsenic: CRC Press; 2001.

[36] Zhang LX, Manard BT, Powell BA, Marcus RK (2015). Preliminary assessment of potential for metal-ligand speciation in aqueous solution via the liquid sampling-atmospheric pressure glow discharge (LS-APGD) ionization source: uranyl acetate. Anal Chem.

[37] Hoegg ED, Williams TJ, Bills JR, Marcus RK, Koppenaal DW (2020). A multi-electrode glow discharge ionization source for atomic and molecular mass spectrometry. J Anal At Spectrom.

[38] Serpe FP, Russo R, Gallo P, Severino L (2013). Method for speciation of organoarsenic in mussels by liquid chromatography coupled to electrospray ionization and QTRAP tandem mass spectrometry. J Food Prot.

[39] Francesconi KA (2002). Applications of liquid chromatography–electrospray ionization-single quadrupole mass spectrometry for determining arsenic compounds in biological samples. Appl Organomet Chem.

[40] McSheehy S, Szpunar J, Lobinski R, Haldys V, Tortajada J, Edmonds JS (2002). Characterization of arsenic species in kidney of the clam tridacna derasa by multidimensional liquid chromatography-ICPMS and electrospray time-of-flight tandem mass spectrometry. Anal Chem.

[41] Larsen BR, Astorga-Llorens C, Florêncio MH, Bettencourt AM (2001). Fragmentation pathways of organoarsenical compounds by electrospray ion trap multiple mass spectrometry (MS6). J Chromatogr A.

[42] Inoue Y, Date Y, Sakai T, Shimizu N, Yoshida K, Chen H (1999). Identification and quantification by LC-MS and LC-ICP MS of arsenic species in urine of rats chronically exposed to dimethylarsinic acid (DMAA). Appl Organomet Chem.

[43] McSheehy S, Mester Z (2003). The speciation of natural tissues by electrospray-mass spectrometry. I: biosynthesized species, As and Se. TrAC, Trends Anal Chem.

[44] Kuehnelt D, Goessler W, Francesconi KA (2003). Nitrogen purity influences the occurrence of As+ ions in high-performance liquid chromatography/electrospray ionization mass spectrometric analysis of four common arsenosugars. Rapid Commun Mass Sp.

[45] Pedersen SN, Francesconi KA (2000). Liquid chromatography electrospray mass spectrometry with variable fragmentor voltages gives simultaneous elemental and molecular detection of arsenic compounds. Rapid Commun Mass Sp.

[46] McSheehy S, Pohl P, Łobiński R, Szpunar J (2001). Complementarity of multidimensional HPLC-ICP-MS and electrospray MS–MS for speciation analysis of arsenic in algae. Anal Chim Acta.

[47] Woller Á, Mester Z, Fodor P (1995). Determination of arsenic species by high-performance liquid chromatography–ultrasonic nebulization–atomic fluorescence spectrometry. J Anal At Spectrom.

[48] Alves MR, Sauer JS, Prather KA, Grassian VH, Wilkins CL (2020). Liquid sampling-atmospheric pressure glow discharge ionization as a technique for the characterization of salt-containing organic samples. Anal Chem.

